# A Novel Treatment of Obstinate Vomiting in Pregnancy

**Published:** 1875-06-15

**Authors:** Edward Copeman


### A NOVEL TREATMENT OF OBSTINATE VOMITING IN PREGNANCY.

By Edward Copeman, M.D., F. R. C. P.

*From the British Medical Journal, May 15, 1875.*

DURING a long professional life, I have had much experience of this troublesome affection, and amongst medicines have found calumba and oxalate of cerium the most beneficial; but these and all other medicines often fail, and the treatment suggested by the following cases, discovered by accident as it were, and never, as far as I know (although nothing is new under the sun), employed before, promises some chance of our being able with more certainty to overcome this very threatening concomitant of pregnancy.

On June 9th, 1874, I was summoned to a lady, thirty-five years of age or thereabouts, to consult with two other practitioners already in attendance. She was about six months gone in pregnancy, and was so reduced by almost incessant vomiting, that great fears were entertained as to her safety. I noticed there was slight uterine action accompanying the sickness, and on examination, I found the os uteri partially dilated so as readily to admit the finger. I thought it right under the emergency to advise bringing on labor without delay; the gentlemen present, however, expressed no little apprehension as to whether or not she would have strength to undergo the effort of parturition on account of the very depressed and exhausted state of her system. They nevertheless concurred in the advisability of the course I recommended, and asked me to perform the operation. I at once dilated the os uteri as much as I could with the finger, and could feel the membranes and the head of the child. I tried to rupture the membranes with a teloscopic female catheter (the only instrument at hand), but they were so flaccid and the head offered so little resistance, the catheter shortening itself also on my making pressure, that I could not succeed; and, thinking it wise to wait awhile before resorting to any other expedient, we retired to another room for further consultation. In about one hour, we saw the patient again, and were surprised to find that a longer period had elapsed without sickness than before; and we again waited, in the hope that she might be able to take a little nourishment, and so be better prepared to undergo any further proceeding. We waited another hour, and another, but there was no return of vomiting; and we spent the rest of the night in watching, during the whole of which time she was improving, and we determined to let well alone. I left her early in the morning, and had a favorable account of her a few days afterwards. There was no return of sickness; she went on to the full period of pregnancy, was then delivered of a healthy child, and made a good recovery.

Two other cases are given, where a similar manipulation resulted in prompt and entire relief.

